# Transcriptome analysis of intestine from alk-SMase knockout mice reveals the effect of alk-SMase

**DOI:** 10.1186/s12935-022-02764-y

**Published:** 2022-11-09

**Authors:** Jiang Zhu, Lingqi Wang, Zhongwu Guo, Tao Zhang, Ping Zhang

**Affiliations:** 1 Medical Laboratory Technology College, Daqing Campus of Harbin Medical University, Daqing, 163319 Heilongjiang China; 2 Department of Laboratory Diagnosis, The fifth Affiliated Hospital of Harbin Medical University, Daqing, 163319 Heilongjiang China; 3 General Surgery, Daqing Oil General Hospital, Daqing, 163319 Heilongjiang China; 4 International School of Public Health and One Health, Hainan Medical University, Haikou, 571199 Hainan China

**Keywords:** Genome-wide, RNA-seq, Alk-SMase, Gene knockout

## Abstract

**Objective:**

Intestinal alkaline sphingomyelinase (alk-SMase) generates ceramide and inactivates platelet-activating factor associated with digestion and inhibition of cancer. There is few study to analyze the correlated function and characterize the genes related to alk-SMase comprehensively. We characterised transcriptome landscapes of intestine tissues from alk-SMase knockout (KO) mice aiming to identify novel associated genes and research targets.

**Methods:**

We performed the high-resolution RNA sequencing of alk-SMase KO mice and compared them to wild type (WT) mice. Differentially expressed genes (DEGs) for the training group were screened. Functional enrichment analysis of the DEGs between KO mice and WT mice was implemented using the Database for Annotation, Visualization and Integrated Discovery (DAVID). An integrated protein–protein interaction (PPI) and Kyoto Encyclopedia of Genes and Genomes (KEGG) network was chose to study the relationship of differentially expressed gene. Moreover, quantitative real-time polymerase chain reaction (qPCR) was further used to validate the accuracy of RNA-seq technology.

**Results:**

Our RNA-seq data found 97 differentially expressed mRNAs between the WT mice and alk-SMase gene NPP7 KO mice, in which 32 were significantly up-regulated and 65 were down-regulated, including protein coding genes, non-coding RNAs. Notably, the results of gene ontology functional enrichment analysis indicated that DEGs were functionally associated with the immune response, regulation of cell proliferation and development related terms. Additionally, an integrated network analysis was shown that some modules was significantly related to alk-SMase and with accordance of previously results. We chose 6 of these genes randomly were validated the accuracy of RNA-seq technology using qPCR and 2 genes showed difference significantly (P < 0.05).

**Conclusions:**

We investigated the potential biological significant of alk-SMase with high resolution genome-wide transcriptome of alk-SMase knockout mice. The results revealed new insight into the functional modules related to alk-SMase was involved in the intestinal related diseases.

**Supplementary Information:**

The online version contains supplementary material available at 10.1186/s12935-022-02764-y.

## Background

Alk-SMase acts with phospholipase C to hydrolyse sphingomyelin (SM) to ceramide, inactivate platelet-activating factor (PAF) and reduce the formation of lysophosphatidic acid (LPA); these effects are all associated with the inhibition of colon cancer [1, 2]. Alk-SMase was discovered in 1969 [3], but the research history of intestinal alk-SMase has been less than 30 years in length. Alk-SMase is expressed in the intestinal mucosa with low activity in the colon and high activity in the jejunum [4]. Alk-SMase, as a novel member of the nucleotide pyrophosphatase and phosphodiesterase (NPP) family, is also called ENPP7 [5]. The hydrolysis of exogenous SM was decreased in alk-SMase KO mice, suggesting an important role of this enzyme in SM hydrolysis in the gut [6].

Recently, some important biological effects of alk-SMase were reported [7]. Researchers purified the protein [8, 9], cloned the gene [5] and found essential roles for the enzyme in phospholipid hydrolysis [4, 10], anti-inflammation [11], anti-tumorigenesis [12], cell proliferation and cholesterol metabolism [13]. In addition, decreased alk-SMase activity was found in colon cancer and colitis [14–16]. Although the role of alk-SMase in the development of colon cancer has been studied, its physiological and pathological significance in the small intestine has not been well explored [3]. In previous studies [5, 6, 8, 9], we found that the functional difference between alk-SMase KO mice and wild-type (WT) mice was due to the deletion of an enzyme. However, the roles of related gene interactions and their transcriptome-level mechanisms have not been elucidated.

Genome-wide transcriptome analysis has become a promising tool to discover global changes in gene profiles under different conditions [17], but the molecular networks and gene interaction mechanisms in alk-SMase KO mice remain largely unknown. In this study, we detected a functional gene set correlated with alk-SMase by transcriptome analysis using a KO model. We performed the first high-resolution RNA sequencing of alk-SMase KO mice and compared them to WT mice. In addition, the transcriptional landscape of alk-SMase KO mice, which had unprecedented resolution, was used to study alk-SMase. Differentially expressed genes (DEGs) between the alk-SMase KO and WT groups were identified with the R package edgeR [18]. A functional enrichment analysis of the DEGs was conducted to understand the biological functions of alk-SMase and to further explore its related genes at the transcriptome level by constructing a gene-to-gene regulatory network that integrated Kyoto Encyclopedia of Genes and Genomes (KEGG) and Protein–Protein Interaction (PPI) data. We explored these biological relationships to further understand the metabolic characteristics and molecular regulation mechanisms of alk-SMase, and the results could provide a theoretical basis for further investigation of the phenotypes and other changes in alk-SMase KO mice.

## Materials and methods

### Animals and samples

The Alk-SMase KO mice were donated by Duan Rui-dong Group. The alk-SMase gene is located on chromosome 11 (Ensembl Gene ID:ENSMUSG 00000046697) in C57BL/6 mice. It was knocked out by the Cre-Loxp system as reported previously. The exon 2 was deleted by Cre recombinase to induce a shift of reading frame and it created an early stop codon and resulted in blocking the translation of the protein. The genotype and phenotype of all mice were assayed by PCR and fecal alk-SMase activity. All mice were housed in the animal facilities at Daqing campus, Harbin Medical University, which were fed commercial standard pellets with free access to water. The mice were anaesthetized by inhaled isoflurane and euthanized by cervical dislocation. All experimental protocols were approved by the Animal Ethics Committee of Harbin Medical University, China. All mice were killed by cervical dislocation under inhaled isoflurane anesthesia and we obtained three matched pairs of intestinal tissue from 3 WT mice and 3 KO mice, respectively. In functional studies, we used alk-SMase knockout mice for experiments. In the DSS-induced colonic inflammatory tumor model, inflammation in alk-SMase KO mice was significantly more intense than in wild type (WT) mice, while in colon cancer models, the number of tumors in KO mice. The degree of malignancy and tumors are also much higher than in WT mice. Our research group have found the important function of alk-SMase in gut, such as anti-inflammatory effect, anti-tumorigenesis, phospholipids hydrolysis and cholesterol metabolism.

### RNA extraction, sequencing and data processing

Total RNA from the middle small intestinal mucosa of every mouse was extracted with the TRIzol method according to the manufacturer’s instructions. The RNA samples were deeply sequenced using the Illumina HiSeq 2500 platform after further clean-up using Qiagen RNeasy Mini columns, and 50 bp single-end reads were generated [19, 20]. Quality control was performed by FastQC (http://www.bioinformatics.babraham.ac.uk/projects/fastqc/), and trim-galore was used to remove the adapter sequences and obtain clean reads. The subsequent processes, including mapping to the reference genome, assembling and gene expression analysis, were carried out by the HISAT2 and StringTie pipeline [21]. HISAT2 was chosen to map the reads to the mouse reference genome (mm10) from the UCSC Genome Browser database, resulting in an alignment rate of 94.18–95.36%.

### Identification of DEGs

The R package edgeR was used to identify DEGs in the intestinal tissue of the knockout group and control group based on fold change > 1.5 (or < 2/3) and FDR < 0.05. The fold change value refers to the multiplier of the gene expression in the treatment group relative to the control group. The fragments per kilobase per million reads (FPKM) values and fold changes of all genes were extracted from the RNA-seq results for further analyses. Three WT samples and three KO samples were compared to find significantly up- and down regulated genes. The heatmaps of the most significantly up regulated and down regulated transcripts were generated with the R command heatmap.2.

### Gene Ontology and KEGG pathway enrichment analyses

In this work, functional enrichment analysis of the DEGs between KO mice and WT mice was implemented using the Database for Annotation, Visualization and Integrated Discovery (DAVID; http://david.abcc.ncifcrf.gov) [22]. Gene Ontology (GO) categories in the biological process ontology and KEGG pathways were identified with a significant threshold p-value ≤ 0.05 and FDR ≤ 0.01.

### Construction of the gene regulatory network and identification of significant genes

In light of the complexity of gene regulation, we expected to find interactions between ENPP7 and DEGs. The protein–protein interaction data were obtained from two PPI databases, Biological General Repository for Interaction Datasets (BioGRID) [23] and Reactome [24]. To obtain more comprehensive interaction information, we used the union data of these two databases as background. In addition, a KEGG network was established based on relationships between genes in the pathway using in-house Perl programs. Eventually, a gene regulatory network was constructed by merging the PPI and KEGG networks. The 97 DEGs were set as seed genes and then mapped into the integrated background network to extract the PPI and KEGG sub-network. The sub-network was composed of these seed genes and the nearby genes (within a one-step distance from the seed genes) in an integrated background network. The network was constructed using Cytoscape 3.3.0 [25], an open-source program used to establish biological networks.

### RT-PCR verification

The mRNA levels of all genes were quantified by real-time PCR. The cDNA was generated using a ReverTra Ace qPCR RT Master Mix kit (Toyobo, China). All primers were obtained from Invitrogen and designed using reference sequences published by the National Center for Biotechnology Information. qPCR was performed on a Roche Light Cycler 480. The reaction volume was 20 µl, including 10 µl of SYBR Green Realtime PCR Master Mix kit (Toyobo, China), 2 µl of diluted cDNA template, and 0.8 µl of 10 µM primers to activate the polymerase, followed by 40 cycles of 95 °C for 15 s and 65 °C for 30 s. The melting curve analysis was performed to confirm that a single product was amplified, and the cycle threshold (CT) was determined by Roche System Software. The relative expression of each mRNA was normalized to the level of the housekeeping gene GAPDH using the 2^−ΔΔCT^ method. Six DEGs were chosen for the experiment. The t-test was used for significance testing between the WT and KO groups, and a p-value ≤ 0.05 was considered significant.

## Results

### Analysis of transcriptome characteristics

**Fig. 1:**
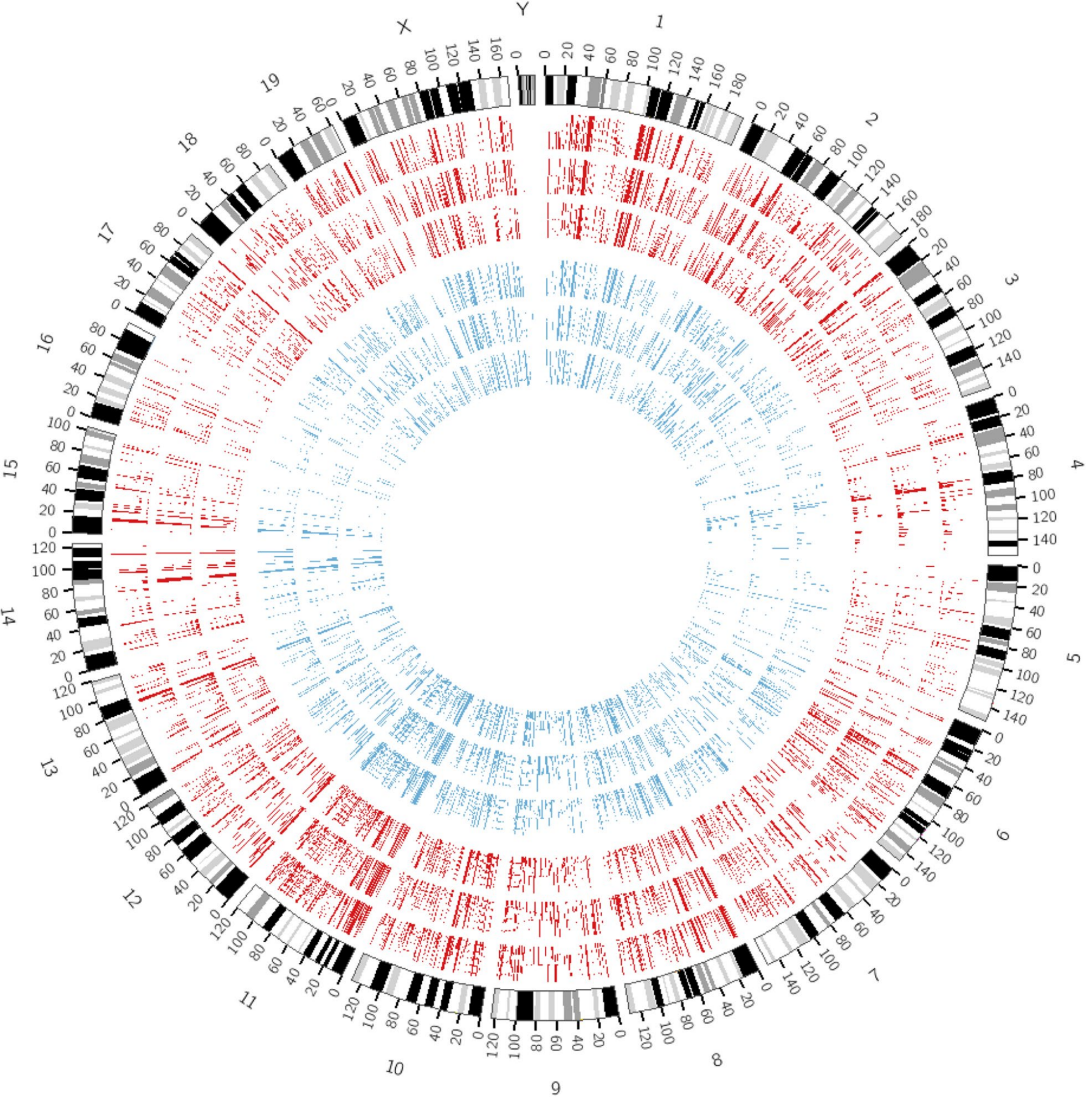
Circos plot of mRNA expression data. Circos v0.62 software was used to visualise global tissue mRNA expression in WT mice and KO mice. Data are shown as a circular map. The three KO samples are shown in the outer three rings, and the inner three rings represent the WT samples. The blue bars represent WT mice, and the red bars represent KO mice

**Fig. 2:**
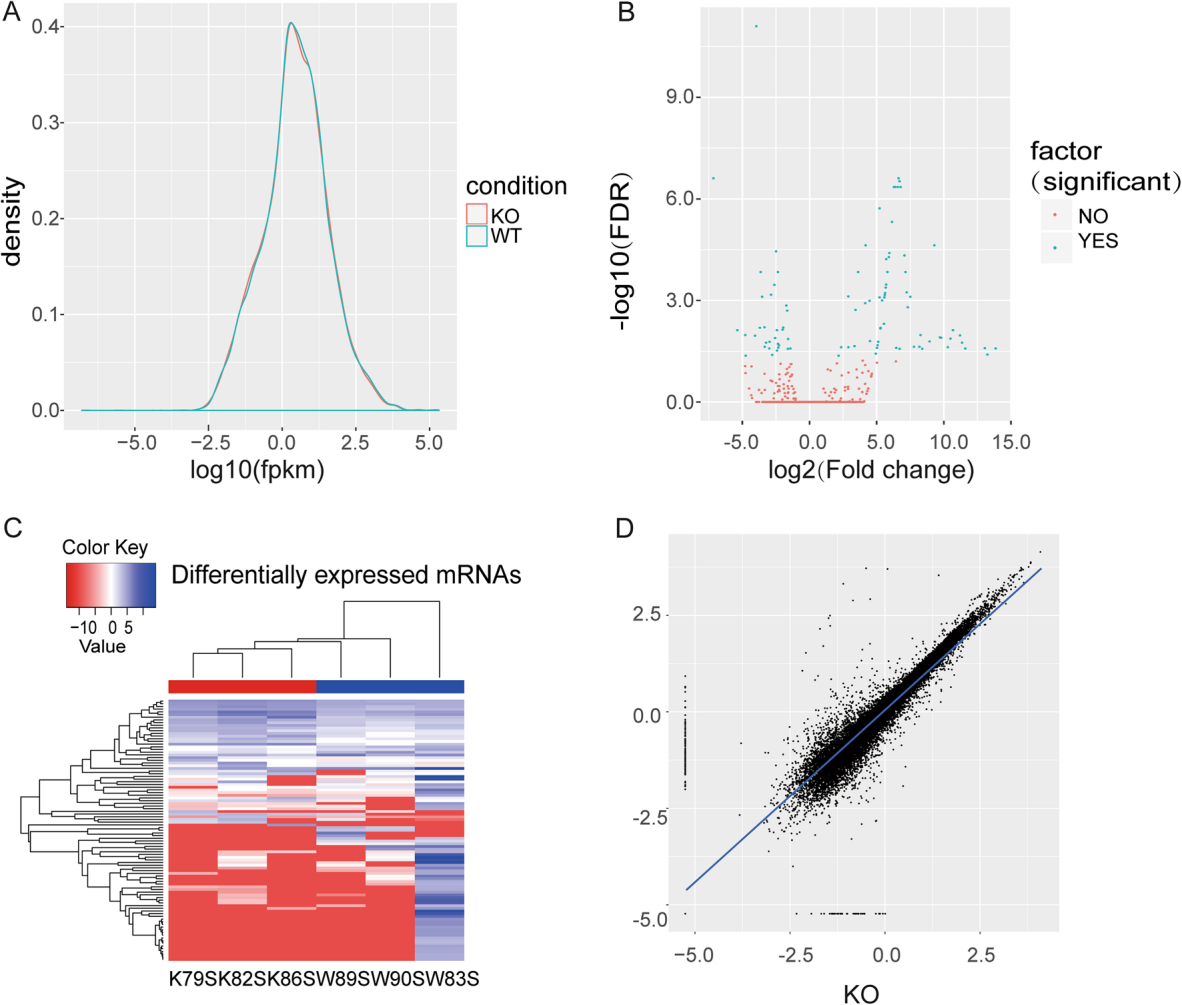
Hierarchical clustering and transcriptome characteristics. **A** Transcript expression distribution density graph of different samples. **B** Volcano plots of the distribution of gene expression. Red dots indicate genes with no statistically significant differences in gene expression, and cyan dots in the figure represent DEGs with statistical significance. **C** Two-way hierarchical clustering of DEGs in KO samples and WT samples. **D** Correlation graph of two types of different samples

Whole transcriptome sequencing was conducted on three matched samples of alk-SMase KO mice and WT mice. In total, approximately 113 million single-end reads were generated by HiSeq 2500 ultra-high-throughput sequencing systems, with an average of 18.9 million reads per sample. After trimming the raw data, the clean data were successfully mapped to the reference genome, and all probable transcripts in the genome were assembled using StringTie. Table 1 lists the read mapping rates for all six samples, which were more than 90%. Quality control of the fastq data produced by the high-throughput sequencers was performed by FastQC software. The expression of all transcripts was normalized by edgeR, and the results were in agreement with a high correlation (Fig. 2D). Although the mRNA expression patterns between the two groups of samples were very similar, numerous differences were observed. The Perl program Circos v0.62 was used to plot genome-wide expression profiles from the intestinal tissue samples from KO mice and WT mice; the data are shown as a circular map in Fig. 1 and a probability density graph in Fig. 2A. Transcripts with low expression (FPKM < 1) in more than 4 samples were removed from the subsequent analysis.

**Table 1:** The overall read mapping rate

Sample	Mapping rate (%)	Input reads	Mapped reads
K79S	95.11	15922606	15143940
K82S	94.85	21424125	20319836
K86S	94.38	12969342	12239110
W83S	95.36	22015543	20994022
W89S	94.18	21904848	20629985
W90S	94.91	16723969	15872812

### Identification of DEGs in KO mice

We performed bioinformatics analyses to investigate the DEGs and functional pathways that characterized the DEGs in our transcriptome. The R package edgeR was used to identify the DEGs. A total of 97 mRNAs were differentially expressed in the intestinal tissue of KO mice compared with that of WT mice, including 32 significantly up regulated and 65 down regulated mRNAs (see Additional file 1). Hierarchical clustering analysis showed that the samples from the KO mice collapsed into one cluster, and those from the WT mice collapsed into the other cluster, suggesting that the expression levels of these DEGs in the KO mice were significantly different from those in the WT mice (Fig. 2C). In Fig. 2B, a heterologous gene expression volcano plot [26] also displays the DEG results. Red dots indicate genes with no statistically significant differences in gene expression, and cyan dots in the figure represent DEGs with statistical significance.

**Fig. 3:**
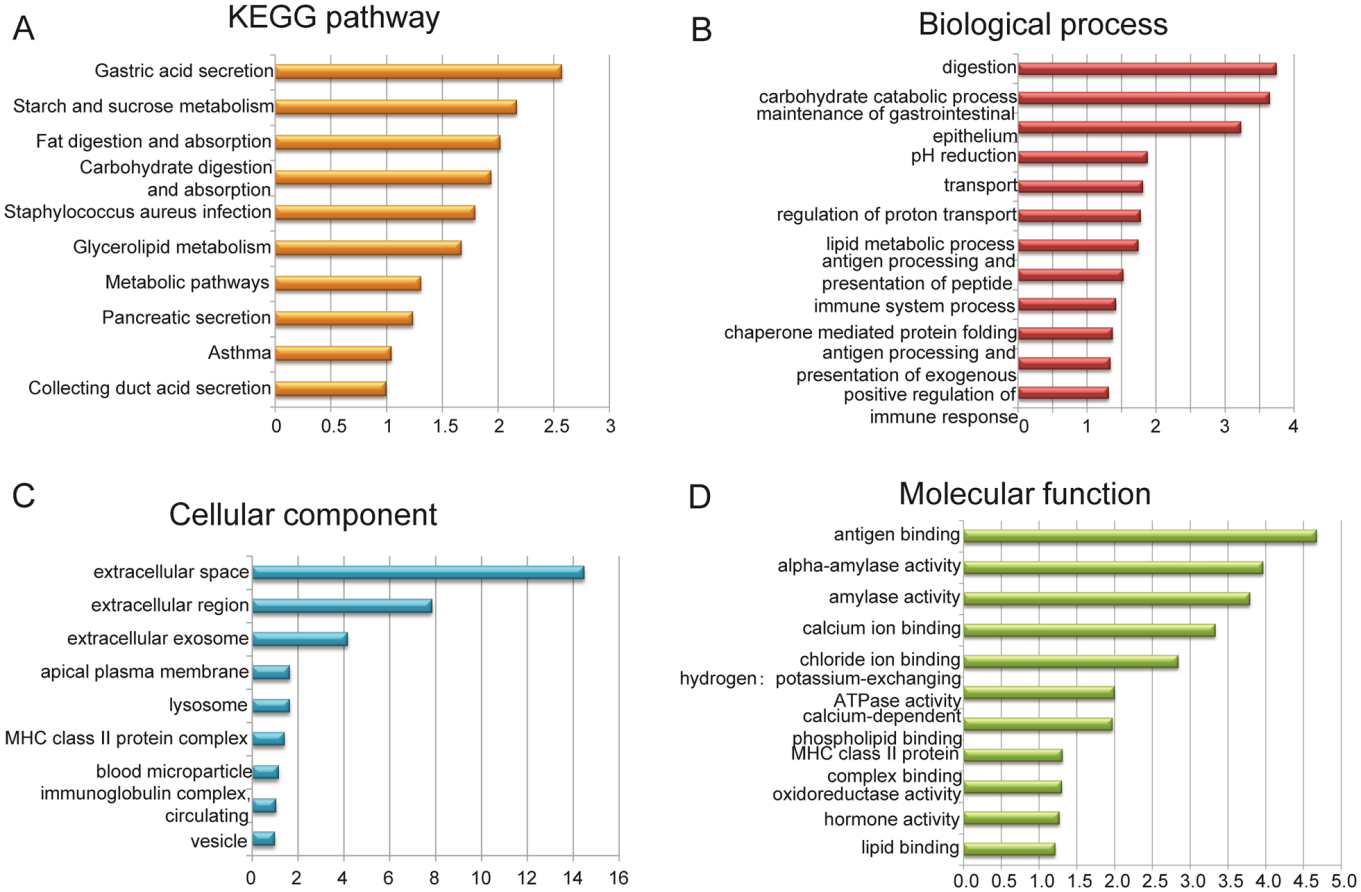
Functional enrichment analysis and enrichment map for DEGs. GO and KEGG enrichment analyses were performed using DAVID with p < 0.05. The results revealed the GO functions and KEGG pathways associated with DEGs. **A** KEGG pathway analysis of DEGs using DAVID. **B** Biological process terms of DEGs using DAVID. **C** Cellular component terms of DEGs using DAVID. **D** Molecular function terms of DEGs using DAVID

To explore the potential biological functions of the DEGs, a functional enrichment analysis was performed for the DEGs using the DAVID functional annotation tool. With a p-value ≤ 0.05 as the threshold, the results indicated that the DEGs were mainly involved in metabolic-process-associated pathways and biological processes including carbohydrate catabolic process, amylase activity, fatty acid binding, and positive regulation of immune response (Fig. 3B–D). These DEGs were enriched in ten pathways, including asthma, gastric acid secretion, starch and sucrose metabolism, fat digestion and absorption, carbohydrate digestion and absorption, glycerolipid metabolism, and metabolic pathways (Fig. 3A).

### Identification of significant genes by EMODE in an integrated PPI and KEGG network

**Fig. 4:**
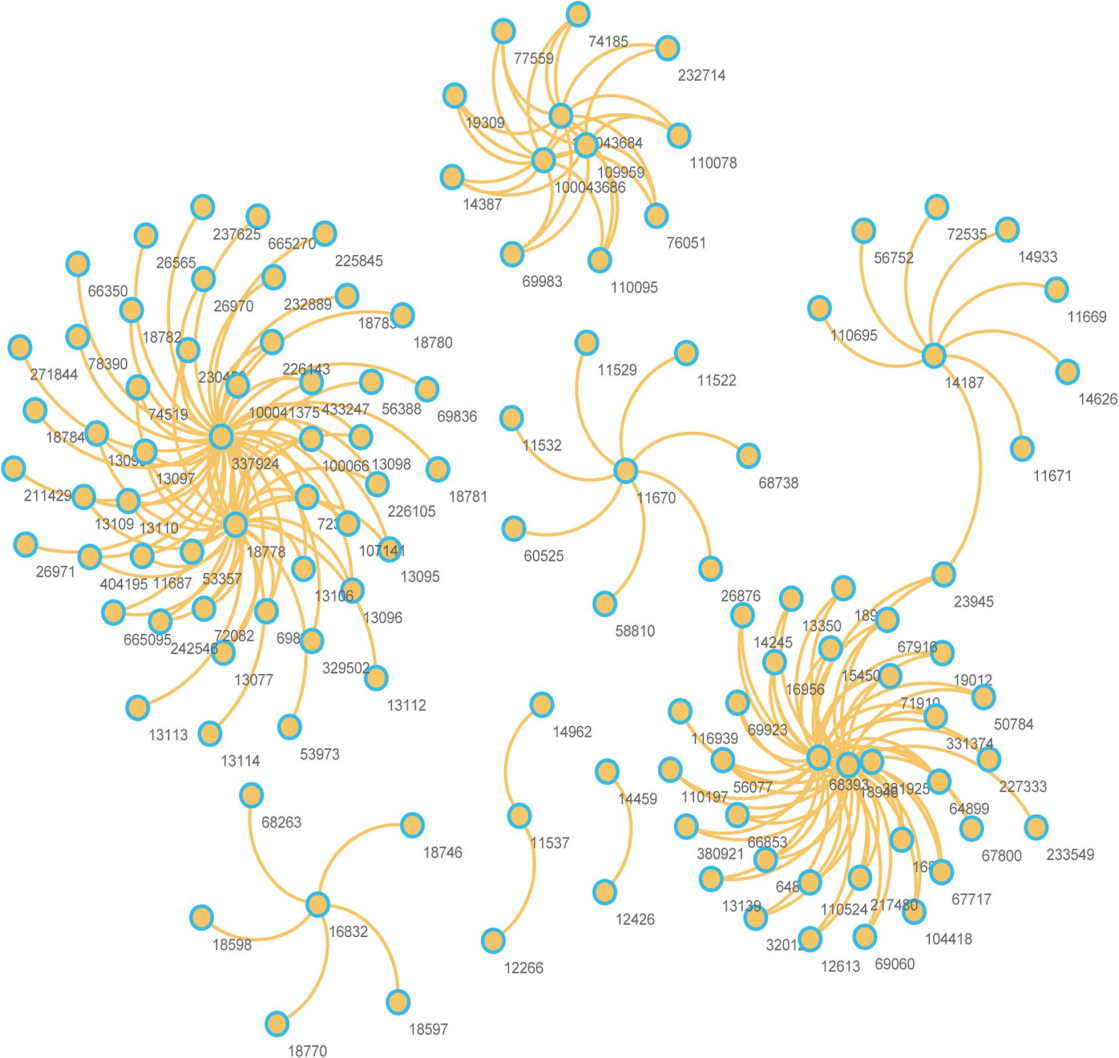
The network was constructed by mapping the DEGs to a PPI and KEGG integrated network. The sub-network was extracted from the PPI-KEGG integrated network by one-step neighbour nodes

As a result of the complexity of gene regulation, we expected to find interaction and regulation among the DEGs in *ENPP7* KO mice. In this study, we selected an integrated PPI and KEGG pathway-based biological network as a background network, in which a total of two sets of mouse PPI data were obtained from BioGRID and Reactome, and the pathways containing DEGs were considered enriched. We mapped 97 seed DEGs into the integrated background network to extract the sub-network. The sub-network was composed of these seed genes and the neighboring genes in the integrated background network (within one step of the seed genes) (Fig. 4).

Considering the genes connected in the network and genes in the same pathway that were thought to have similar and biologically related functions, dividing these highly connected genes into groups by network analysis may be useful for potential functional processes in a manner complementary to standard differential expression analysis. In the sub-network, a small number of nodes had high degrees, while many had low degrees. The hub genes in this sub-network included *Cyp3a44, Pnliprp1, Mogat1, Pla2g1b, Amy2a4, Amy2a3, Amy2a5, Akr1b8, Aldh3a1* and *Ldhb* (Table 2).

**Table 2:** Hub genes in the PPI and KEGG network

Gene	Regulation	Degree
Cyp3a44	Up	44
Pnliprp1	Up	34
Mogat1	Down	33
Pla2g1b	Down	31
Amy2a4	Down	9
Amy2a3	Up	9
Amy2a5	Down	9
Akr1b8	Up	8
Aldh3a1	Up	7
Ldhb	Up	7

### Validation of DEGs by real-time PCR

**Fig. 5:**
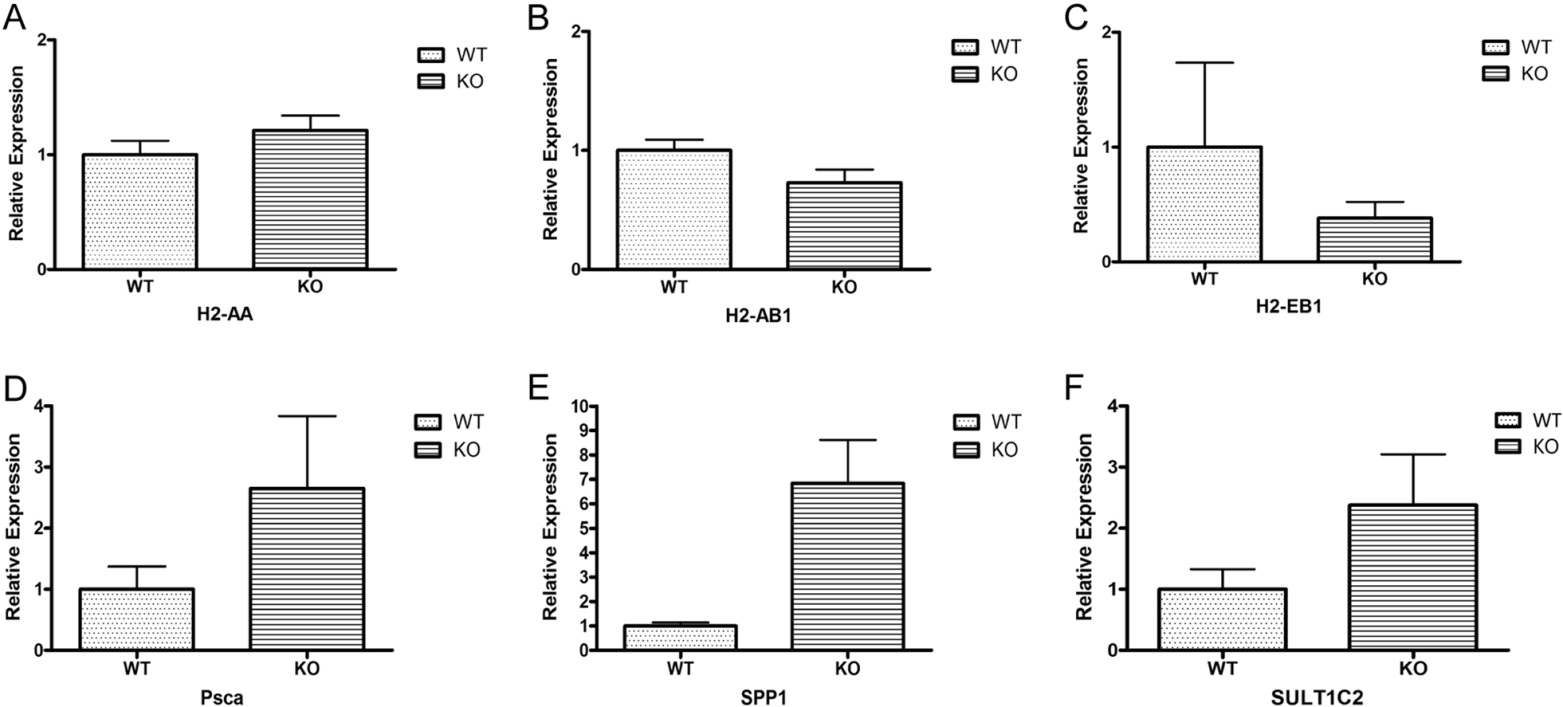
Results of RT-PCR performed on RNA extracted from WT mice and ENPP7 KO mice. **A**–**F** Represent H2-AA, H2-AB1, H2-EB1, Psca, SPP1, and SULT1C2, respectively

To validate the accuracy of the RNA-seq data, we further analyzed the expression of six randomly selected transcripts by RT-PCR. As shown in Fig. 5, the results of the RT-PCR experiment suggested that the expression of all 6 transcripts was consistent with the RNA-seq data, and the *H2-AB1* and *SPP1* genes showed significant differences by t-test (P < 0.05) [27], the additional file shows this in more detail (see Additional file 2). However, the differential expression analysis included three biological replicates, and a subset of the transcripts showed opposite expression patterns between replicates, indicating individual differences. In addition, although the mean expression values in the figures showed a noticeable difference, it was not statistically significant.

## Discussion

Some biological effects of alk-SMase have been reported, including the essential roles ofthis enzyme, including anti-inflammatory and anti-tumorigenesis effects, phospholipid hydrolysis and cholesterol absorption [28]. However, the mechanism of action of alk-SMase in the intestine remains poorly understood. Here, we performed a comprehensive analysis to investigate the interactions and relationship between alk-SMase (*ENPP7*) and other genes. The objective to find out the key genes of alk-SMase interactions in the intestine. Notably, decreased alk-SMase activity has been reported in colon cancer and colitis [29]. Although the role of alk-SMase in the development of colon cancer has been studied, the biological significance and interactions of this enzyme have not been well studied in the intestine. Transcriptome analysis is an effective way to study and understanding the basis of phenotypic variation in different conditions [30]. Here, we used transcriptome profile analysis to reveal DEGs. A total of 97 DEGs were identified, and these genes were related to immune-related diseases and lipid metabolism and carbohydrate catabolic processes. It has been found that some pathway and GO terms are closely related to lipid and glucose metabolism and cell differentiation [31, 32], and proliferation [33]. We expected that the knock-out model of alk-SMase gene could be used to analyze the interactions between alk-Smash and other genes, or the regulation of related genes, so as to play a protective role in the intestinal mucosal barrier, as we have found in the experiments, or other biological functions. The absence of NPP7 caused alterations in gene expression in the intestine transcriptomes in KO mice. The alteration of intestinal function is caused by the loss of expression of intestinal basic sphingomyelin, which affects bile acid metabolism, bile acid intestinal hepatic circulation and the expression of other related genes. Among the DEGs, a large number of genes are worthy of subsequent analyses, and transcriptome sequencing is a very effective method to select functional gene sets for related pathways. For example, *Muc1* is highly expressed in colon cancer and has been reported as a new target antigen for tumour vaccines. In addition, serum *Muc1* is a new indicator that may be involved in tumour invasion, which can assist in the early diagnosis of colon cancer [34]. Meanwhile, Annexin A13 (*ANXA13*) is a surface protein of *Lactobacillus reuteri* [35]. Mucosal adhesion proteins are bound to the surfaces of the intestinal epithelial cells by *ANXA13* and *PALM*. *ANXA13* is a receptor-like molecule and an immunoregulatory factor with anti-inflammatory effects [36]. Together, the DEGs that we identified maintain the intestinal mucosal barrier function of alk-SMase in the intestine.

Numerous transcription changes involving complex gene pathways, including metabolism, immune response and inflammation, have been described. RNA sequencing allows rapid quantification of gene expression in small number of tissue, whether in whole or in a targeted way. In order to verify the potential biological relationships between DEGs, we constructed a network based on PPI and pathway information. By arranging the first ten degrees, ten central genes were identified from the subnet. Up regulation of *Mogat1* conceivably mediates hepatic steatosis and insulin resistance through increasing intracellular diacylglycerol content [37], consistent with our results. PLA2G1B mediates lipid absorption, and PLA2G1B-derived metabolic products contribute to cardiometabolic diseases, including obesity, hyperinsulinaemia, and atherosclerosis [38]. In the future, we can further analyze the functions of these genes.

To examine the accuracy of the RNA-seq data, we analyzed the expression of six transcripts by quantitative real-time PCR. RT-PCR results showed that all 6 transcription levels were consistent with RNA-seq results, and there were significant differences between H 2-AB 1 and SPP 1 genes through T-test. We investigated H2-AB1 in the literature and found that H2-AB1 was differentially expressed in endothelial cell RNA isolated from control and hyperlipidaemic prelesion mice by expression array analysis [39]. The level of H2-AB1 was highly increased in allergic rhinitis, and it was associated with the pathogenesis of allergic rhinitis [40]. Another differentially expressed transcript, secreted phosphoprotein 1 (SPP1), a highly phosphorylated protein, plays important roles in physiological processes such as inflammatory responses, calcification, organ development, carcinogenesis response and immune cell function [41, 42], suggesting that SPP1 is also an important marker in the alk-SMase KO model and may participate in functional and phenotypic effects.

A comprehensive analysis of the experiment showed some exciting results. A large number of immunoglobulin-related genes, which have been found to affect immune function in the mucosal barrier, were among the DEGs. Insulin growth factor binding protein was also a gene of interest. Whether this enzyme is related to insulin metabolism or diabetes merits further experiments. A large number of lipid-metabolism-related enzymes are associated with alk-SMase, and tight junction proteins provide important antibodies for our analysis of mucosal barrier function. Overall, the RNA-seq analysis provided valuable guidance for future experiments.

## Conclusions

In this study, DEGs was successfully identified and characterized in the alk-SMase gene KO model of mice. A total of 97 DEGs, including Mogat1, Pla2g1b, and Amy2a4, were identified across the whole genome. Our results showed that these DEGs were enriched in metabolism-related pathways. Our study provides the first systematic genome-wide analysis of the alk-SMase knockout model, alk-SMase plays an important role in the in intestinal sphingomyelin hydrolysis. The expression levels of SPP1 and H2-AB1 were effected in intestine tissues lacking alk-SMase expression.

## Supplementary Information

**Additional file 1**. The list of differentially expressed genes

**Additional file 2**. The original experimental data of qPCR

## Data Availability

All data generated or analysed during this study have been submitted to the Genome Expression Omnibus (GEO) database, the accession number is GSE114608 (https://www.ncbi.nlm.nih.gov/geo/query/acc.cgi?acc=GSE114608). To review GEO accession GSE114608: Go to https://www.ncbi.nlm.nih.gov/geo/query/acc.cgi?acc=GSE114608. Enter token qtezmuculhihfgn into the box.

## Abbreviations

alk-SMase: Alkaline sphingomyelinase
BioGRID: Biological general repository for interaction datasets
DAVID: Database for annotation visualization and integrated discovery
GO: Gene Ontology
KEGG: Kyoto encyclopedia of genes and genomes
KO: Knoc kout
qPCR: Quantitative real-time polymerase chain reaction
WT: Wild type
