# Correction: Transcriptome analysis of intestine from alk-SMase knockout mice reveals the effect of alk-SMase

**DOI:** 10.1186/s12935-022-02795-5

**Published:** 2022-11-29

**Authors:** Jiang Zhu, Lingqi Wang, Zhongwu Guo, Tao Zhang, Ping Zhang

**Affiliations:** 1 Medical Laboratory Technology College, Daqing Campus of Harbin Medical University, Daqing, 163319 Heilongjiang China; 2 International School of Public Health and One Health, Hainan Medical University, Haikou, 571199 Hainan China; 3 Department of Laboratory Diagnosis, The Fifth Affiliated Hospital of Harbin Medical University, Daqing, 163319 Heilongjiang China; 4 General Surgery, Daqing Oil General Hospital, Daqing, 163319 Heilongjiang China

## Correction: Cancer Cell International (2022) 22:344 https://doi.org/10.1186/s12935-022-02764-y

In this article, the affiliation details of all the authors were incorrectly assigned. The correct affiliations are given in this correction:

Correct order of author affiliations and notation as followed:

Jiang Zhu^1†^, Lingqi Wang^1,3†^, Zhongwu Guo^4^, Tao Zhang^1^ and Ping Zhang^2^*

^1^Medical Laboratory Technology College, Daqing Campus of Harbin Medical University, Daqing 163319, Heilongjiang, China.

^2^International School of Public Health and One Health, Hainan Medical University, Haikou 571199, Hainan, China.

^3^Department of Laboratory Diagnosis, The fifth Affiliated Hospital of Harbin Medical University, Daqing 163319, Heilongjiang, China.

^4^General Surgery, Daqing Oil General Hospital, Daqing 163319, Heilongjiang, China.

The original article [1] has been corrected.
